# Home practice for robotic surgery: a randomized controlled trial of a low-cost simulation model

**DOI:** 10.1007/s11701-023-01688-7

**Published:** 2023-08-02

**Authors:** Rachel K. Wile, Riley Brian, Natalie Rodriguez, Hueylan Chern, Jason Cruff, Patricia S. O’Sullivan

**Affiliations:** 1 School of Medicine, University of California, San Francisco, 533 Parnassus Ave, San Francisco, CA 94143 USA; 2 Department of Surgery, University of California, San Francisco, 513 Parnassus Avenue, S-321, San Francisco, CA 94143 USA; 3 Department of Obstetrics/Gynecology-Female Pelvic Medicine & Reconstructive Surgery, Marshfield Clinic Health System, Marshfield, WI 54449 USA

**Keywords:** Robotic surgery, Robotic surgery simulation, Surgical education, Home simulation model, da Vinci skills simulator, SimNow

## Abstract

Pre-operative simulated practice allows trainees to learn robotic surgery outside the operating room without risking patient safety. While simulation practice has shown efficacy, simulators are expensive and frequently inaccessible. Cruff (J Surg Educ 78(2): 379–381, 2021) described a low-cost simulation model to learn hand movements for robotic surgery. Our study evaluates whether practice with low-cost home simulation models can improve trainee performance on robotic surgery simulators. Home simulation kits were adapted from those described by Cruff (J Surg Educ 78(2): 379–381, 2021). Hand controllers were modified to mimic the master tool manipulators (MTMs) on the da Vinci Skills Simulator (dVSS). Medical students completed two da Vinci exercises: Sea Spikes 1 (SS1) and Big Dipper Needle Driving (BDND). They were subsequently assigned to either receive a home simulation kit or not. Students returned two weeks later and repeated SS1 and BDND. Overall score, economy of motion, time to completion, and penalty subtotal were collected, and analyses of covariance were performed. Semi-structured interviews assessed student perceptions of the robotic simulation experience. Thirty-three medical students entered the study. Twenty-nine completed both sessions. The difference in score improvement between the experimental and control groups was not significant. In interviews, students provided suggestions to increase fidelity and usefulness of low-cost robotic home simulation. Low-cost home simulation models did not improve student performance on dVSS after two weeks of at-home practice. Interview data highlighted areas to focus future simulation efforts. Ongoing work is necessary to develop low-cost solutions to facilitate practice for robotic surgery and foster more inclusive and accessible surgical education.

**Supplementary Information:**

The online version contains supplementary material available at 10.1007/s11701-023-01688-7.

## Introduction

Robotic surgery is a positive, enduring innovation that surgeons are increasingly integrating into surgical practice for complex minimally invasive procedures [1]. As robotic surgery has gained relevance to multiple surgical specialties, and particularly in general surgery, urology, and gynecology, it has become an important component of surgical education [2]. Pre-operative simulated practice offers surgical trainees the opportunity to learn robotic surgery outside of the operating room, allowing them to improve their skills without impacting patient safety [2, 3].

Various robotic surgery simulators currently are available, including the da Vinci Skills Simulator (dVSS; Intuitive Surgical Inc., Sunnydale, California, USA) with the SimNow learning program, the dV-Trainer (dVT; Mimic Technologies, Inc., Seattle, Washington, USA), RobotiX Mentor (RM; 3D Systems, Littleton, Colorado, USA), SimSurgery Educational Platform Robot (SEP; SimSurgery, Oslo, Norway), and Robotic Surgical Simulator (RoSS; Simulated Surgical Systems LLC, San Jose, California, USA) [4, 5]. These simulators provide a similar experience to actual robotic surgery by using a robotic console and virtual reality (VR) technology [4]. Practice using VR simulators has been shown to help trainees develop skills that translate to improved operating room performance in robotic surgery [3, 6, 7].

A previous study has shown that dVSS outperforms the other two most commonly used VR simulators (dVT and RM) for content validity [5]. dVSS uses the da Vinci Xi surgeon console along with the computer learning program SimNow to provide the user with 47 basic training exercises and 33 “life-like” simulated robotic-assisted surgical procedures to practice [8]. The basic skill tasks allow the trainee to learn essential robotic surgery skills, including Endowrist manipulation, camera positioning, clutching, needle control and driving, energy, and dissection [8, 9]. Additionally, the program provides the trainee with a score for each task at the end of the exercise based on their path length, which is termed “economy of motion,” as well as the time to complete the exercise [8]. Any penalties, such as instrument collisions or excessive force, are also factored into the final score [5, 8]. A total score of greater than 90% indicates “mastery” of the task [5].

While simulation training sessions that provide hands-on practice for robotic surgery are effective, simulators are expensive and not readily accessible to many community surgeons and students [10, 11]. Furthermore, as multiple modalities of performing surgery have emerged—including open, laparoscopic, endovascular, and robotic—trainees face increasing demands on their training time. Developing an accessible, easily reproducible robotic surgery simulation model for on-demand practice could provide trainees with an additional tool to prepare for robotic surgery.

One prior report from Cruff [10] described a low-cost home simulation model for learning the hand movements required for robotic surgery [10]. The author detailed the creation of hand controllers that mimic the feel of the master tool manipulators (MTMs) on the da Vinci robot, allowing for accessible practice in any location at any time [10]. However, this descriptive paper did not assess outcomes related to the use of this model [10]. The purpose of our study is to evaluate whether practice with a low-cost home simulation model can improve trainees’ performance on the robotic surgery simulators. Additionally, we sought to understand novices’ experiences practicing with the home simulation model to evaluate if it is a user-friendly and effective tool for trainees learning robotic surgery.

## Materials and methods

### Development of home simulation kits

**Fig. 1:**
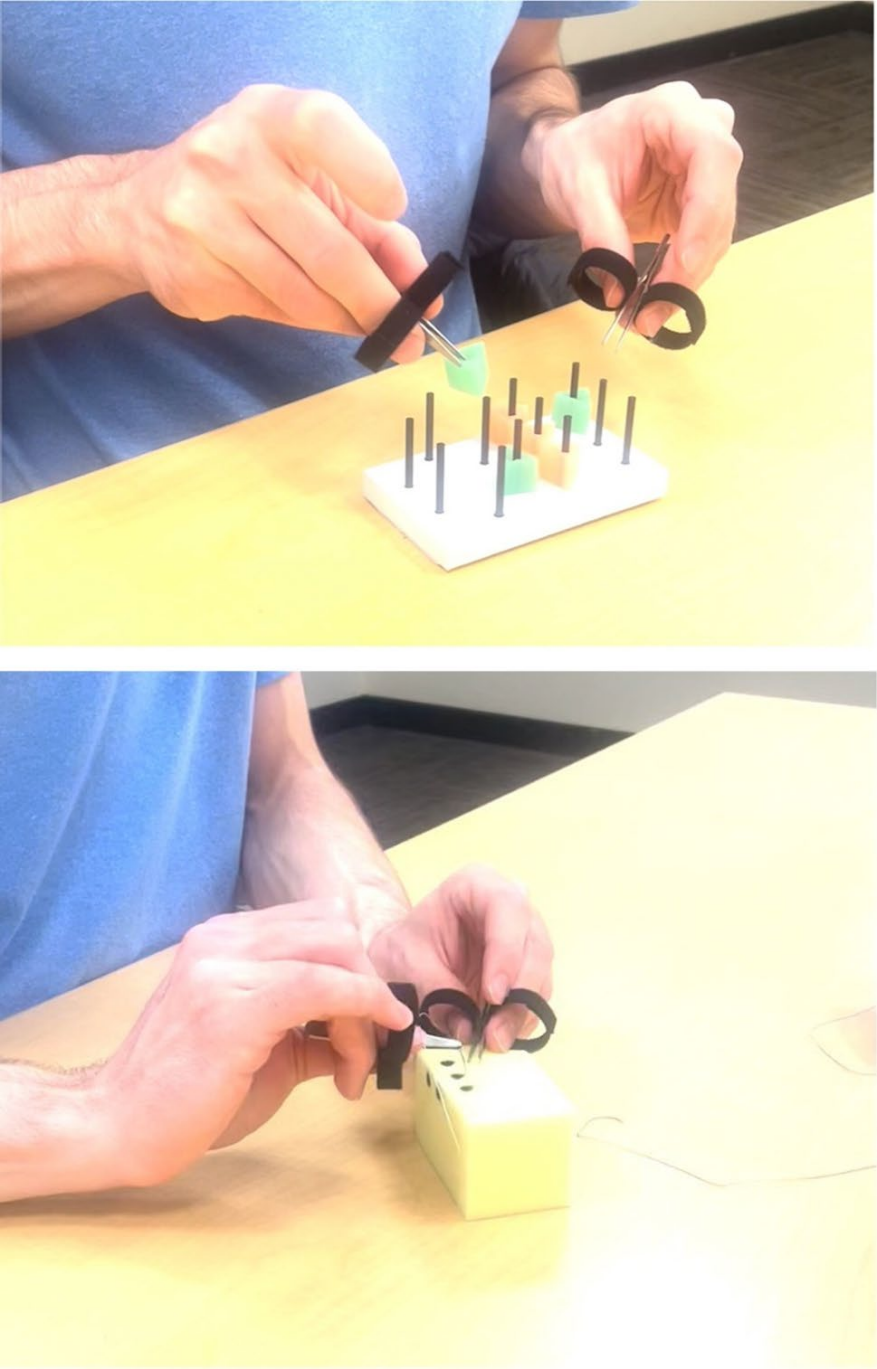
Home simulation kits including the hand controllers, peg transfer board, and sponge

The controllers in the home simulation kits were designed to mimic the MTMs on the dVSS and were modeled from those described by Cruff [10]. Five different models of the hand controllers were assessed by experienced robotic surgeons at our institution who have interacted with the robotic hand controls over hundreds of robotic operations. The final model was made by taking a fine point tweezer and affixing two small Velcro loops as finger slots (Fig. 1). The ends of the metal tweezers were cut to widen the surface area to make it easier to grasp objects and to match the MTMs more closely. In addition to the two controllers, the home simulation kit also included a peg transfer board, sutures, and a sponge.

The peg transfer board allowed trainees to practice an exercise that mimicked the Sea Spikes 1 task on the SimNow program. The Sea Spikes 1 task requires the trainee to virtually grasp a series of rings and place them on the color concordant cone [8]. To model this exercise with the home simulation kits, the participants in the experimental group were instructed to use the model controller in their left hand to grasp each of the six triangles on the pins oriented in a circle on the peg transfer board. Then, they were to transfer each triangle individually to the controller in their right hand and place it down on the pins oriented in a rectangle on the right side of the pegboard. This exercise was adapted from its laparoscopic correlate described in the Fundamentals of Laparoscopic Surgery (FLS) [12].

The home simulation kits also included a sponge and suturing needles. These materials allowed the participants to practice needle driving with the hand controllers in a manner intended to mimic the Big Dipper Needle Driving task on SimNow. The Big Dipper Needle Driving task is a virtual suturing exercise on a foam suture pad, in which the trainee must drive the needle through a sequence of holes [8]. A series of dots were drawn on the sponge included in the home simulation kit to closely resemble the holes on the virtual suturing pad of the Big Dipper Needle Driving task. Using the home simulation kit, participants were able to practice driving the needle in and out of the different marks on the sponge to replicate the same pattern as the task in the simulator.

### Study design and participants

Volunteer medical students who were novices to robotic surgery were recruited to participate in this study. The University of California, San Francisco (UCSF) Institutional Review Board determined this study to be exempt (IRB22-36266). All participants completed a pre-study survey on general demographics and factors that may affect their performance on the robotic simulator exercises, including video game experience [13] and surgical experience (Supplemental File 1). All participants then completed a baseline session on dVSS using SimNow, in which they completed two exercises (Sea Spikes 1 and Big Dipper Needle Driving).

After completion of this baseline SimNow session, participants were randomly assigned to either receive a robotic home simulation kit with instructions for practice or to receive no home simulation kit. Participants in the experimental group received their home simulation kit immediately following the baseline SimNow session and were instructed to practice with the hand controllers using the peg transfer board and suture supplies for 15 min each day for 2 weeks. Studies have shown that learning a task can be done more effectively when practice sessions are shorter and more frequent [14–16]. The practice time of 15 min every day for two weeks was chosen based on the efficacy of the distributed practice model and expectations of student commitment to practice.

**Fig. 2:**
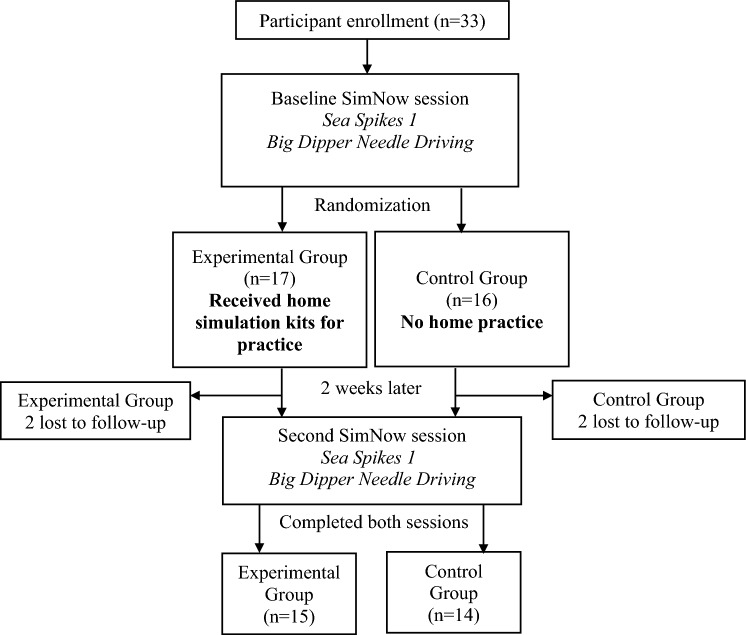
Flowchart of study design

Furthermore, these students received instructional video supplements that outlined proper technique for the peg transfer and needle driving exercises to facilitate correct practice with the home simulation kits. Students in the experimental group were also provided a daily log to detail the specific activities and the time they spent practicing. Two weeks after the baseline SimNow session, all participants returned for another session using the SimNow program on dVSS to repeat the Sea Spikes 1 and Big Dipper Needle Driving tasks (Fig. 2).

### Main study parameters

After each trial of a task, SimNow produces a score report that provides an overall score (out of 100) for the trainee’s performance. In addition to the overall score for each task, the score report is also broken up into various components. The pre-specified performance parameters examined in this study included: (1) time taken to complete the exercise, (2) economy of motion, and (3) penalty sub-score.

### Statistical analysis

Descriptive statistics regarding the demographics of the participants, including their year in medical school, gender, age, video game use, handedness, and experience in the OR, as well as with laparoscopic surgery and robotic surgery, were assessed using R version 4.1.3. The groups were compared on these demographics using Fisher’s exact test and *t* tests. A paired *t* test was used to compare the experimental group participants’ scores from the first session and the second session for both the sea spikes 1 task and the big dipper needle driving task. The same was completed for the control group. An ANCOVA controlling for pre-score was conducted using SPSS version 28 to compare the differences in SimNow final scores adjusting for baseline between students who did (experimental group) and who did not (control group) practice with the home simulation kit. Significance level was set at 0.05.

### Qualitative interviews

Following the second SimNow session, all the students from the experimental and control groups participated in semi-structured debriefing interviews with RW regarding their experience using the robotic surgery simulator during the first and second simulator sessions. As a medical student, RW was chosen to conduct the interviews to allow for the most open and frank discussion with interviewees. Participants in the experimental group were asked additional questions to learn about their experience using the home simulation kit (Supplemental File 2). All interviews were recorded with participant consent and responses were transcribed. The primary goal of these interviews was to understand how specific skills, movements, and knowledge gained from the use of the home simulation kit applied to the robotic surgery simulator exercises on SimNow. The secondary goal of the interviews was to identify common challenges that participants faced when practicing with the home simulation kit and to find any improvements that could be made to the design.

All the transcripts were reviewed by two readers (RW and RB) independently and codes were identified using an inductive approach. The readers came to a consensus on developing the codebook. All transcripts were then double-coded, and the codes were reconciled. Themes were identified among coded data. In this analysis and to identify themes, RW brought her perspective as a medical student with no robotic surgery experience, and RB brought his perspective as a surgical resident with robotic surgery experience.

## Results

### Demographics

Overall, 33 participants completed the first dVSS session, of whom 29 participants completed both dVSS sessions (Fig. 2). Of these 29 participants, 15 participants (51.7%) received a home simulation kit and instructions for practice initially after the baseline robotic simulation session and 14 participants (48.3%) did not. Based on practice logs, experimental group participants practiced with the home simulation kits for a mean time of 138 min (66% of the practice goal), with a standard deviation of 86.3 min. Baseline characteristics of the control and experimental group did not differ significantly (Table 1).

**Table 1:** Participant demographics and baseline characteristics

Variable	Cohort, *n* = 29	Home simulation kit group (experimental), *n* = 15	No home simulation kit group (control), *n* = 14	*p* value
Age, yrs (SD)	24.86 (1.84)	25.14 (2.07)	24.57 (1.60)	0.42
Gender, *N*				
Male	16	8	8	NC*
Female	12	6	6	
Non-Binary	1	1	0	
Year in medical school, *N*				
1st	14	7	7	NC*
2nd	10	7	3	
Higher level	5	1	4	
Handedness, *N*				
Right	25	12	13	0.60
Left	4	3	1	
Played video games regularly, *N*				
Yes	19	8	11	0.25
No	10	7	3	
Assisted in OR, *N*				
Yes	9	4	5	0.70
No	20	11	9	
Experience in robotic surgery, *N*				
Yes	7	4	3	1.0
No	22	11	11	
Experience in laparoscopic surgery, *N*				
Yes	9	4	5	0.70
No	20	11	9	

*Not calculated because sample size was too small to meet statistical assumptions

### SimNow performance

At the second trial, the experimental group improved their mean overall score by 30.26 points with a standard deviation of 25.82 points for sea spikes 1 (Table 2). The average improvement in the control group’s overall score was 14.28 points with a standard deviation of 27 points. The difference between the two groups was not significant by an ANCOVA controlling for pre-score.

**Table 2:** Scores for baseline and follow-up SimNow sessions

	Group Receiving Home Simulation Kits (*n* = 15)		*p* value^1^	Control Group (*n* = 14)		*p* value^1^
	Average Score Session 1	Average Score Session 2		Average Score Session 1	Average Score Session 2	
Sea Spikes 1						
Overall SimNow Score (SD)	37.67 (24.60)	67.93 (16.22)	**0.00046**	52.43 (20.37)	66.71 (20.16)	0.070
Economy of motion, cm (SD)	474.3 (134.0)	349.9 (62.50)	**0.0036**	386.16 (67.72)	341.26 (51.19)	0.057
Time to completion, s (SD)	284.1 (128.7)	185.9 (37.78)	**0.0033**	231.23 (61.57)	199.5 (66.27)	0.11
Penalty subtotal (SD)	– 21.13 (27.58)	– 6.07 (7.80)	0.060	– 10.36 (8.14)	– 6.00 (8.31)	0.127
Big Dipper Needle Driving						
Overall SimNow Score (SD)	1.27 (4.91)	11.53 (14.89)	**0.016**	3.64 (7.30)	22.79 (22.24)	**0.0020**
Economy of motion, cm (SD)	889.0 (214.8)	696.2 (165.9)	**0.0020**	765.5 (186.6)	615.1 (124.8)	**0.0034**
Time to completion, s (SD)	792.9 (226.9)	610.59 (185.8)	**0.0011**	753.1 (227.4)	550.34 (149.3)	**0.00016**
Penalty subtotal (SD)	– 31.00 (16.85)	– 25.60 (11.79)	0.075	– 24.71 (10.61)	– 16.07 (5.38)	**0.0024**

^1^Bolded values are significant with p<0.05.

For the Big Dipper Needle Driving task, both the experimental and control groups showed significant improvements during the second trial in their overall score, economy of motion, and time to completion. There were no significant differences between the experimental and control groups in the outcomes for Big Dipper Needle Driving between the first and second dVSS sessions. Furthermore, there were no significant associations between changes in score and practice time.

### Qualitative interviews

All 29 students who returned for the second robotic simulator session completed the post-session interview immediately following the second task. Through the participant interviews, we identified three main themes of student experience: (1) Novices encounter initial challenges with the robotic simulator that improve with exposure, (2) Practice with the home simulation kit impacts the robotic simulator experience, and (3) The fidelity of the home simulation kit could be improved (Table 3).

**Table 3:** Qualitative results summary

Themes	Subthemes	Exemplar quotes
Novices encounter initial challenges with the robotic simulator that improve with exposure	1. Lack of Familiarity – Orienting to the robot – Understanding goals and rules 2. Lack of technical skills – Body positioning – Instrument control – Needle handling 3. Visuospatial challenges – Depth perception	Familiarity—“I wasn’t fully cognizant of all the rules and also just conceptually figuring out how to use the clutch, when to use it, and how to orient yourself while you’re using it was a little bit tricky for me.” Technical skills—“The first time it was hard to maintain both of the arms of the robot in a distance so they don’t collide with themselves, that was challenging. I think I did that a couple times and…I was penalized.” Technical skills—“Just getting used to the clutch was really hard.” Technical skills—“I had a really hard time figuring out the orientation for the needle.” Visuospatial skills—“I feel like just getting used to the disconnect between what you’re seeing and then where your hands are, because not seeing your actual hands while you’re doing something.”
Practice with the home simulation kit impacts the robotic simulator experience	1. Technical skills – Needle handling – Wristed movements – Ambidexterity 2. Familiarity	Technical skills—“The needle threading for the home kit was pretty good because you get the dexterity of how to go about putting the needle in one hole and getting it out the other one.” Technical skills—“[The kit helped] needle control and kind of determining which ways I needed to twist my wrist and my fingers in order to get [the needle] to drive through, which definitely translated over here [at the robotic simulator].” Technical skills—“Just being a little bit more ambidextrous with how I did the needle driving activity and trying to use the other hand because I’m predominantly right–handed and I think for the first time I was using predominantly my right hand, but I was…more conscious of which hand I was using [during the second session].” Familiarity—“I learned how to go about the needle driving activity most efficiently, how to angle my hands so that I could basically just put the needle through very easily and seamlessly.”
The fidelity of the home simulation kit could be improved	1. Model design transferability – No clutch on home model – More resistance than robot 2. Mechanical challenges	Model design—“If you’re actually threading the needle through the real sponge, you’re going to feel resistance, but here [on the robotic simulator], you don’t feel anything.” Mechanical challenges—“The tips of the tweezers didn’t have much grip either, and so you’re… pressing really, really hard to hold the needle in place, because otherwise it was just slipping out of place. So I guess maybe swap to a different type of tweezer that might have more grip.”

#### Novices encounter initial challenges with the robotic simulator that improve with exposure

Students experienced challenges upon initial exposure to the robotic simulator in three areas: (1) familiarity, (2) technical skills, and (3) visuospatial skills. These challenges framed subsequent experiences with home simulation.

Many participants found it difficult to orient themselves to the robot and the tasks, as well as understanding the rules and goals of each exercise.

“I would say at first the most challenging is just being intentional with all of my hand movements. I wasn’t fully cognizant of all the rules and also just conceptually figuring out how to use the clutch, when to use it, and how to orient yourself while you’re using it was a little bit tricky for me.” (Experimental Participant #18)

Even after they became familiar with the simulator, the participants noted that they struggled with using the robot to perform the technical skills required to complete the SimNow tasks, including body positioning, instrument control, and needle handling. Concerning body positioning, students reported difficulties determining how they should orient their arms in physical space under the console of the machine to most effectively and comfortably complete each task on the simulator.

“The first time it was hard to maintain both of the arms of the robot in a distance so they don't collide with themselves, that was challenging. I think I did that a couple times and…I was penalized.” (Control Participant #1)

Regarding instrument control, most students specified they had initial difficulties with effectively using the clutch on the MTMs to complete the tasks.

“Just getting used to the clutch was really hard. I usually forgot to engage the clutch and so I would twist myself into like 35 positions, which made it hard.” (Experimental Participant #27)

Students also found needle handling difficult in their initial exposure to the robotic simulator.

“I had a really hard time figuring out orientation for the needle…so that I could insert it correctly, and that it came out exactly where I wanted it to, because I would put the needle into the sponge and pretty much have no idea how to turn my hand or where it was basically in the surface.” (Control Participant #16)

Students initially experienced challenges with their visuospatial skills, such as depth perception. Many students found it hard to perceive the environment through the VR robotic console and felt they did not have a precise idea of the movements they were making.

“I feel like just getting used to the disconnect between what you’re seeing and then where your hands are, because not seeing your actual hands while you’re doing something.” (Control Participant #22)

Participants in both the experimental and control groups identified that their abilities changed from the first SimNow session to the second SimNow session. During the second session, they were more familiar with the interface and activities, felt more comfortable with basic skills, and had improved understanding of the depth perception and use of visual cues.

#### Practice with the home simulation kit impacts the robotic simulator experience

Within the experimental group, participants attributed a variety of experiences during the second SimNow session to their practice with the home kit, including both technical skills and understanding of the robotic console.

In terms of technical skills, participants felt that practicing needle manipulation with the controllers provided with the home simulation kits was useful to develop their dexterity and improve their needle handling and wristed movements.

“[The home simulation kit was] helpful to do the motion; just have your fingers in the right place and kind of pick things up and put things down. The sponge was helpful in judging the needle orientation, and the hand motions you need to do the scoop.” (Experimental Participant #12)

“[The kit helped] needle control and kind of determining which ways I needed to twist my wrist and my fingers in order to get [the needle] to drive through, which definitely translated over here [at the robotic simulator].” (Experimental Participant #17)

Others stated that the home simulation kit helped improve their ambidexterity, a helpful skill for many tasks on the robotic surgery simulator.

“Just being a little bit more ambidextrous with how I did the needle driving activity and trying to use the other hand because I’m predominantly right-handed and I think for the first time I was using predominantly my right hand, but I was…more conscious of which hand I was using [during the second session].” (Experimental Participant #18)

When probing specifically about how the home simulation kit affected the participants’ haptic feedback, or sense of touch, on the robotic simulator, students highlighted the differences between the haptic sensation in the home kit and that of the robotic simulator.

“It's definitely a different haptic feeling. I felt like it was harder to drive the needle through the sponge than it is to drive the needle through the fake robotic sponge. But, just having the haptic feedback was helpful and that is something you get with the home kit.” (Experimental Participant #12)

Finally, participants also communicated that the home simulation kit improved their sense of familiarity with the tasks and allowed them to strategize to complete the tasks more efficiently.

“I learned how to go about the needle driving activity most efficiently, how to angle my hands so that I could basically just put the needle through very easily and seamlessly.” (Experimental Participant #18)

“I think that I was a little bit like, I wouldn’t say slower, but more kind of methodical, and that's something that came from the practice.” (Experimental Participant #4)

#### The fidelity of the home simulation kit could be improved

While participants found the home kits to be helpful in improving their skills and comfort with the robotic simulator, they also had some suggestions for refining the kits for future use. Their suggestions focused on model design transferability to the robotic simulator, as well as on the mechanical challenges they experienced.

Regarding model design transferability, despite the usefulness of the sponge to learn to judge needle orientation and to practice the wrist motions to drive the needle, it had a different feeling than the robotic simulator. In general, participants expressed that using a sponge for the needle driving activity in the home kit produced more resistance than the robotic simulator. Students reported that this difference made it challenging to understand exactly how much pressure should be used when driving the needle on the robotic simulator. Some suggested using another material with less resistance to better mimic the virtual foam and its lack of haptic feedback on the robotic simulator.

“If you’re actually threading the needle through the real sponge, you’re going to feel resistance, but here [on the robotic simulator], you don’t feel anything.” (Participant #14)

Participants also suggested that it would be helpful to include something on the home kit that would closely resemble the clutch of the robotic simulator.

“Maybe put some type of pad for the clutch, because this basically felt slightly different…I got used to pressing with my finger curled up and when I was like, ‘Oh wait, where’s the clutch?’ and I had to re-find the clutch.” (Experimental Participant #26)

Regarding mechanical challenges, participants noted that certain parts of the home kit were less comfortable to use or needed to be reinforced to prevent any breakage, interfering with their ability to use the practice kits as directed. Particularly, many students mentioned the difficulties they experienced with the stiffer grip of the tweezers used to design the hand controllers in the home simulation kits.

“The tips of the tweezers didn’t have much grip either, and so you’re… pressing really, really hard to hold the needle in place, because otherwise it was just slipping out of place. So I guess maybe swap to a different type of tweezer that might have more grip.” (Experimental Participant #17)

## Discussion

Given the need to provide practice opportunities to prepare learners to use new surgical technologies, this study evaluated home practice for robotic surgery. Overall, low-cost robotic surgery home simulation kits did not lead students to better performance on dVSS compared to control students, though interview data highlighted the potential of home simulation and future directions for simulator development. The score increase in both groups between simulation sessions likely reflects task familiarity on dVSS gained during the first session. Challenges with physical fidelity of the home robotic simulation kits, as discussed by many participants in interviews, may have limited additional skill gains in the home practice group. Furthermore, many participants did not meet the practice goal, likely because home practice was unsupervised and unscheduled. Additionally, the duration of practice was self-reported, which may have been inaccurate and did not account for how correctly the participants practiced. Finally, since medical students are not expected to perfect their robotic surgical skills, they may have less motivation to practice compared to, for example, surgical residents who will be expected to master these skills.

For future iterations of the home simulation kit, participants noted the importance of addressing the haptics of the model and choosing appropriate practice tasks. While this feedback from medical students will be important in planning future studies, it would also be helpful to include surgical residents with more experience in further trials. Prior experience with the robot may allow trainees to focus home practice on the most useful skills, while also practicing with better technique, leading to greater overall improvement.

There remains a need for a low-cost simulation model to facilitate engagement and accessible practice for robotic surgery. Many trainees do not have formal robotic surgery simulation training curricula in place at their institutions [17, 18]. Common barriers to including robotic simulation in programs are cost and access to simulator facilities [17]. Although low-cost models may not replace VR simulators, prior work has shown the promise of simulators representing a range of physical fidelities, including in robotic surgery [19, 20]. Simulation may be most educational and cost-effective when a coordinated progression of simulator fidelity is thoughtfully introduced [21]. Optimizing a low-cost robotic simulation model could provide a helpful bridge to practice for early trainees who have more limited access to the robotic simulators.

This study begins to address this need by describing important considerations for low-cost robotic simulator development. Improvements to model design transferability, such as fine-tuning the resistance of the controllers or including a clutch, could potentially lead to more efficacious practice and greater improvements in performance. It was apparent that a number of students in the experimental group showed strong motivation to practice their skills with the home simulation kits. Some students spent several hours practicing with the home kits and found them to be an excellent way to engage with robotic surgical skills. Having additional time to practice at home can be an important component of learning when there is limited student exposure to robotic surgery [22, 23]. Thus, further refining this intervention may increase early trainees’ engagement with robotic surgery. In the future, it may also be more helpful to focus on developing the specific technical skills necessary for success in robotic surgery, rather than replicating the specific simulated tasks. Previously, expert robotic surgeons have deconstructed robotic surgery into seven key elements: pick and place, two-handed transfer, wrist manipulation, camera control, clutching, suturing, and energy use [24, 25]. An ideal home simulation kit can provide specific practice for the technical skills of pick and place, two-handed transfer, and wrist manipulation. Future work with the home kits can specifically target these transferable skills to potentially lead to greater improvement in performance.

In conclusion, the home simulation kits will need to be further refined to better represent the robotic surgery controllers and the practice tasks should be modified to optimize user skill acquisition in the future. However, work with home simulation kits represents the beginning of a needed innovation in robotic surgery. The development of creative, low-cost solutions to practice surgical techniques is essential to reach more potential trainees and foster more inclusive and accessible surgical education.

## Supplementary Information

Below is the link to the electronic supplementary material.

Supplementary file1 (DOCX 15 KB)

## Data Availability

The data that support the findings of this study are available on request from the corresponding author, RW.

## Abbreviations

MTMs: Master tool manipulators
dVSS: da Vinci skills simulator
VR: Virtual reality
SS1: Sea spikes 1
BDND: Big dipper needle driving
FLS: Fundamentals of laparoscopic surgery
