# Supplementary material for: Home practice for robotic surgery: a randomized controlled trial of a low-cost simulation model
Source: J Robot Surg. 2023 Aug 2;17(5):2527–36. doi: 10.1007/s11701-023-01688-7 (PMC10492874; doi:10.1007/s11701-023-01688-7)
Supplement: Supplementary file 1 — Supplementary file1 (DOCX 15 KB) [file 11701_2023_1688_MOESM1_ESM.docx]

**Supplemental File 1** Pre-study questionnaire on general demographics and baseline characteristics

Demographics

1. Year in medical school:
2. Gender: (male, female, transgender male/transman/FTM, transgender female/transwoman/MTF, gender queer, non-binary, gender not listed decline to answer)
3. If you selected “gender not listed,” please specify:
4. Age:
5. Do you play or have you ever played video games regularly (including PC/mac, gaming console, smartphone/tablet)? Y/N
6. When was the last time you regularly played video games?
   1. Within the last 3 months
   2. Within the last year
   3. Within the last 5 years
   4. When I was a child
   5. I have never played video games
7. On average how much time did you spend playing video games?
   1. < 1 hr/week
   2. 1-5 hr/week
   3. 6-10 hr/week
   4. > 10 hr/week
   5. N/A
8. Do you consider yourself right-handed, left-handed, or ambidextrous? R/L/ambidextrous
9. Please state your hand preference for the following activities: (always left hand, most often left hand, both hands/doesn’t matter, most often right hand, always right hand)
   1. Writing
   2. Throwing an object
   3. Brushing teeth
   4. Eating with a spoon
10. What OR-related experiences do you have?
    1. Shadowed in the OR – Y/N
    2. Assisted in the OR – Y/N
    3. Worked as a nurse/scrub technician/rep for a company that sells surgical tools, robots, etc. – Y/N
11. Have you had any experience in laparoscopic surgery? (y/n)
    1. How many cases have you observed?
    2. How many cases have you participated in?
    3. How did you participate? (check all that apply)
       1. Observed
       2. Closed skin
       3. Driving the camera
    4. Comments/explanation regarding your laparoscopic experience:
12. Have you had any experience in robotic surgery? (y/n)
    1. How many cases have you observed?
    2. How many cases have you participated in?
    3. How did you participate? (check all that apply)
       1. Observed in room
       2. Observed through the console
       3. Helped with patient at bedside
       4. Helped change robotic instruments
       5. Closed skin
    4. Comments/explanations regarding your robotic experience:
13. What specialty are you interested in pursuing? (anesthesia, general surgery, internal medicine, neurology, neurosurgery, OB/GYN, orthopedic surgery, otolaryngology, pediatrics, plastic surgery, psychiatry, urology, other, undecided)

**Supplemental File 2** Interview Guide

Questions for both experimental and control group:

1. What was challenging about the in-person robotic simulation practice during the first round?
   1. How about today (during second round)?
2. What specific skills were you able to improve between the first and second in-person robotic simulation sessions?

Questions for experimental group only:

1. What did you learn from practice with the home simulation kit?
   1. What specific skills did the kit help you improve? (examples: fine motor skills, instrument control, needle handling)
   2. Haptics include the sense of touch that is received when operating. Do you feel that the home practice helped you develop this haptic feeling for what was happening on the robotic simulator?
   3. How did you apply what you learned from the home practice to the in-person robotic simulation practice?
2. What was challenging about the home robotic simulation practice?
   1. Are there any changes you would make to the home simulation kit to make it more accurately representative of the robotic surgery simulator or more user friendly?
